# The Biodegradability and *in Vitro* Cytological Study on the Composite of PLGA Combined With Magnesium Metal

**DOI:** 10.3389/fbioe.2022.859280

**Published:** 2022-03-15

**Authors:** Xue Wang, Hui Sun, Mang Song, Guangqi Yan, Qiang Wang

**Affiliations:** ^1^ School and Hospital of Stomatology, China Medical University, Shenyang, China; ^2^ Liaoning Provincial Key Laboratory of Oral Diseases, Shenyang, China

**Keywords:** PLGA, magnesium, biomaterial, bone defect, osteogenesis

## Abstract

The main goal of this study was to develop a novel poly (lactic-co-glycolic acid) (PLGA) composite biodegradable material with magnesium (Mg) metal to overcome the acidic degradation of PLGA and to investigate the cytocompatibility and osteogenesis of the novel material. PLGA composites with 5 and 10 wt% Mg were prepared. The samples were initially cut into 10 mm × 10 mm films, which were used to detect the pH value to evaluate the self-neutralized ability. Murine embryo osteoblast precursor (MC3T3-E1) cells were used for *in vitro* experiments to evaluate the cytotoxicity, apoptosis, adhesion, and osteogenic differentiation effect of the composite biodegradable material. pH monitoring showed that the average value of PLGA with 10 wt% Mg group was closer to the normal physiological environment than that of other groups. Cell proliferation and adhesion assays indicated no significant difference between the groups, and all the samples showed no toxicity to cells. As for cell apoptosis detection, the rate of early apoptotic cells was proportional to the ratio of Mg. However, the ratios of the experimental groups were lower than those of the control group. Alkaline phosphatase activity staining demonstrated that PLGA with 10 wt% Mg could effectively improve the osteogenic differentiation of MC3T3-E1 cells. In summary, PLGA with 10 wt% Mg possessed effective osteogenic properties and cytocompatibility and therefore could provide a wide range of applications in bone defect repair and scaffold-based tissue engineering in clinical practice.

## Introduction

Bone defects resulting from performing procedures for the head–neck cysts or tumors are common clinical problems. Therefore, synthesis of biomaterials with biocompatibility and controlled degradability to repair bone defects has always been a hot topic in the maxillofacial field (Phasuk et al., 2018; Zhang et al., 2019).

Poly (lactic-co-glycolic acid) (PLGA) possesses good biocompatibility, suitable mechanical strength, and controlled degradability, and is widely used in pharmaceutical and medical engineering materials (Omezli et al., 2015; Martins et al., 2018). PLGA, which has mechanical and degradable characteristics that can be regulated by varying the lactic acid (LA) to glycolic acid (GA) ratio (Makadia and Siegel, 2011; Gentile et al., 2014), has been used to produce biodegradable sutures and orthopedic fixation plates according to these characteristics (Hutmacher, 2000; Goth et al., 2012). Moreover, PLGA can inhibit the infiltration of macrophages, which are beneficial for bone regeneration and repairs (Ren et al., 2021). However, PLGA degradation can lead to an acidic environment in local tissues, which affects bone tissue regeneration and slows down the degradation rate of the material (Fu et al., 2000; Zhao et al., 2021).

In recent years, magnesium (Mg) and its alloys as metal biomaterials have attracted considerable attention because of their lightweight, bone-like mechanical, and osteoconductive properties (Landi et al., 2008; Hort et al., 2010; Farraro et al., 2014; Chakraborty Banerjee et al., 2019). Some authors have reported that Mg and its alloys have been used in orthopedic appliances in patients with bone defects (Staiger et al., 2006). The advantages also include antibacterial properties because of the alkaline environment with a local high pH value after Mg degradation. However, Mg degrades rapidly *in vivo*, and uncontrolled degradation increases the pH value of the local environment, which affects the process of osteogenic differentiation (Jana et al., 2021). With regard to the uncontrolled degradation of Mg, other studies have investigated coating bulk Mg alloys with PLGA, poly (L-lactic acid), or poly (ε-caprolactone) to control the degradation rate of the alloy (Wong et al., 2010; Guo et al., 2011; Ostrowski et al., 2013; Johnson et al., 2016). Furthermore, Yu et al. develop a composite bioactive scaffold composed of polylactide-coglycolide and tricalcium phosphate incorporating osteogenic, bioactive magnesium metal powder (Yu et al., 2019). These coatings decrease short-term degradation rate and increase cell attachment and viability. The cellular studies showed that the composites with higher Mg particle concentration showed higher cell viability, cytocompatibility, migration, and osteogenesis differentiation. Most recently, many authors reported similar coating techniques and high-quality coating materials on their articles (Chen et al., 2018; Tang et al., 2022).

Based on the physicochemical complementary characteristics of PLGA and Mg, we aimed to develop a novel PLGA/Mg composite biodegradable material. We hypothesized that 1) Mg degradation products can neutralize the acidic byproducts in the process of decomposing PLGA and 2) the PLGA/Mg composite biomaterial can promote osteogenesis. To identify a better ratio of PLGA and Mg, 5 and 10 wt% Mg were added to PLGA to form the composite material. Immersion tests and pH value monitoring were performed to evaluate the degradability and self-neutralizing ability of the composite material. Cell proliferation, attachment, apoptosis, and osteogenesis assays were performed *in vitro*.

## Materials and Methods

### Synthesis of PLGA

The PLGA copolymers with a molar ratio of 50/50 were synthesized as follows. Equal molar amounts of L-lactide (LLA) and glycolide were charged in a polymerization tube, and the catalyst solution (0.2 mol, 1/5,000 eq.) was then added to the reaction mixture. The tube was purged with dry nitrogen and kept under vacuum for 2 h to remove all volatiles. The tube was heat-sealed under vacuum, and copolymerization was carried out at 130 ± 2°C for 3 days. After the reaction, the tube was broken, the copolymer was dissolved in chloroform, further purified in ice-cold methanol, and dried under vacuum to a constant weight.

### Preparation of PLGA/Mg Composite Biomaterials

PLGA (10 g) was dissolved in dichloromethane at a ratio of 1:5 g/ml, and pure Mg powder (50 μm average diameter) provided by Institute of Metal Research, Chinese Academy of Sciences was then added to the polymer solution to form composites with 5 and 10 wt% Mg metal. The solution was vortexed for 30 min before casting into a polytetrafluoroethylene dish and left to air dry at 4°C for 3 days. After solvent evaporation, the films were dried to a constant weight under vacuum. The dried films were cut into strips 10 mm long and 10 mm wide.

### pH Value Monitoring

The samples were divided into three groups: P group, P5% Mg group, and P10% Mg group. Each sample was cut into 10 mm × 10 mm films and then placed into a phosphate-buffered saline (PBS) solution. The surface for each sample was immersed in the PBS buffer solution. The change in the pH of PBS solution was detected every day. The test included 7 days of continuous pH value monitoring. Finally, the pH value of each sample was calculated for three times, and the average value was recorded for future analysis.

### *In Vitro* Experiments

#### Cell Culture

Murine calvarial preosteoblast (MC3T3-E1) cells were cultured in Alpha modified Eagle medium (α-MEM; Hyclone Corporation, United States) supplemented with 10% fetal bovine serum (Clark, Virginie Ledoyen, United States), 100 U/mL penicillin, and 0.1 mg/ml streptomycin. The cells were stored in an incubator at 37°C with 5% CO_2_. Trypsin (0.25%; Sigma Corporation, United States) was used to digest and passage the cells. MC3T3-E1 cells were used to assess cell cytotoxicity, adhesion, apoptosis, and osteogenic regeneration.

#### Cell Cytotoxicity Test

Cell cytotoxicity tests were performed using the CCK-8 kit (US Everbright Inc., Silicon Valley, United States). Cells were incubated in 96-well cell culture plates (Corning, NY, United States) at a density of 2 × 103 cells/well. After 24 h of cell attachment, the medium was replaced with 100 μL of the extracts, and the control groups were replaced with normal culture medium. Each group had five biological replicates. The 96-well cell culture plates were incubated at 37°C in a humidified atmosphere with 5% CO_2_ for 24 h, 3, 5, and 7 days. Subsequently, 100 μL of α-MEM with 10% CCK-8 was added to each well, and the 96-well cell culture plates were incubated with CCK-8 solution at 37°C for 4 h. The spectrophotometric absorbance of the samples was measured using a microplate reader (Infinite M200, Tecan, Austria) at 450 nm. All tests were repeated three times. Relative growth rate (RGR) was used to evaluate the biocompatibility of the composite material. The formula for calculating RGR was as follows: RGR = OD_e_/OD_c_×100%. OD_e_ is the average OD value of the experimental group. OD_c_ is the average OD value of the control group. Cell toxicity grade (CTG) was based on the value of RGR, referring to the standard United States Pharmacopeia (Jin et al., 2013). A material was considered non-toxic when the RGR value of the sample was greater than 80 and the CTG grade was 0 or 1 according to the criterion (Vincent et al., 2018).

#### Cell Apoptosis

Flow cytometry (Becton Dickinson Corporation, United States) was performed to determine the apoptosis rate of MC3T3-E1 cells cultured with samples in the three groups. Each group included three samples, which were cleaned and sterilized. The samples were then placed in 12-well plates for the subsequent procedure. MC3T3-E1 cells with 50–60% confluence in a culture flask were seeded in 12-well plates with sterilized samples at a density of 5 × 10^4^ cells/well for 24 h. The medium was then replaced every 48 h. Annexin V-FITC/PI double staining was performed at 1, 3, and 7 days after planting the cells using the Annexin V-FITC/PI double staining kit (US Everbright Corporation, United States) according to the manufacturer’s instructions. Early apoptotic cells were localized in the lower right quadrant of the dot-plot graph.

#### Cell Adhesion Activity Assay

To determine the cell adhesion activity of the samples in the three groups, an adhesion assay was performed. The samples used for this assay in each group were initially sterilized before detection. The MC3T3-E1 cells were then seeded onto the samples in 12-well plates at a density of 5 × 10^3^/well for 1, 2, and 3 days. Subsequently, the samples were washed with phosphate buffer solution three times, followed by fixation with 2.5% glutaraldehyde for 4 h. After washing with PBS solution, the samples were dehydrated using alcohol with 30, 50, 75, 95, and 100% concentrations in sequence. Rhodamine-phalloidin and DAPI staining were performed to visualize the cytoskeleton and cell nuclei, respectively. Fluorescence microscopy (Nikon, Tokyo, Japan) was used to observe the results.

#### Alkaline Phosphatase Activity Staining

ALP staining was used to test mineralization activity (Golub et al., 1992). MC3T3-E1 cells were seeded onto the sample surfaces at a density of 5 × 10^4^ cells/well in 12-well plates. After 24 h, the medium was replaced with osteogenic medium (α-MEM culture medium supplemented with 10 mM beta-glycerophosphate [Sigma, St. Louis, MO], 50 mg/ml ascorbic acid [Sigma], and 10^−8^ M dexamethasone [Sigma]), which was changed every 2 days for 7 and 14 days. The samples were washed twice with PBS solution, followed by fixation with 4% paraformaldehyde for 10 min. BCIP/NBT staining was performed to detect osteogenic activity according to the manufacturer’s instructions for the BCIP/NBT ALP staining kit (Beyotime, Shanghai, China). The results were observed using a fluorescence microscope (Nikon, Japan).

### Statistical Analysis

All experiments were performed at least in triplicate. Data were analyzed using GraphPad Prism software (version 6.0; GraphPad Software, San Diego, CA, United States) and are shown as mean ± standard deviation. Statistical analysis was performed using SPSS 21.0 software. One-way analysis of variance followed by Tukey’s post-test was performed in this study. Statistical significance was set at *p* < 0.05.

## Results and Discussion

### Characterization of PLGA

**FIGURE 1:**
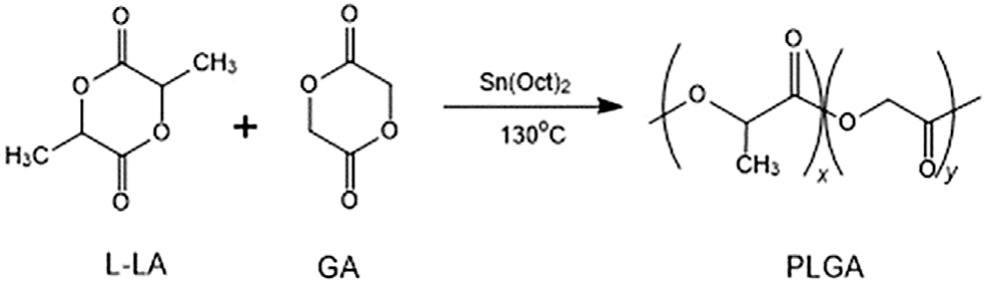
The synthetic route of PLGA.

PLGA was synthesized by bulk ring-opening copolymerization of LLA and GA in the presence of SnOct_2_ as a catalyst at 130°C. The synthesis of PLGA is illustrated in Figure 1. ^1^H NMR (δ, ppm from TMS in CDCl_3_):1.52 (−CH_3_, LA unit), 5.20 (−CH<, LA unit), and 4.85 (−CH_2_−, GA unit). The LA/GA ratios in the PLGA copolymer were calculated by comparing the ratios of absorbances at 5.20 ppm (−CH<, LA unit) and 4.85 ppm (−CH_2_−, GA unit), which were found to be 46.4/53.6 in the copolymer. The larger fraction of GA in the copolymer than that charged in the monomer feed was due to the higher reactivity of GA in comparison with LLA.

The molecular weight and polydispersity of the PLGA copolymers were determined using GPC, showing a polydispersity of 1.70 and a molecular weight of 1.05 × 10^5^ g/mol. The thermal properties of the PLGA copolymer were determined using DSC and TGA. The TGA thermogram of PLGA showed that the polymer began to degrade at 272°C, and the DSC studies showed that the PLGA copolymer was amorphous with only one glass transition temperature of 32.6°C.

### Characterization of PLGA/Mg Composite Biomaterials

**FIGURE 2:**
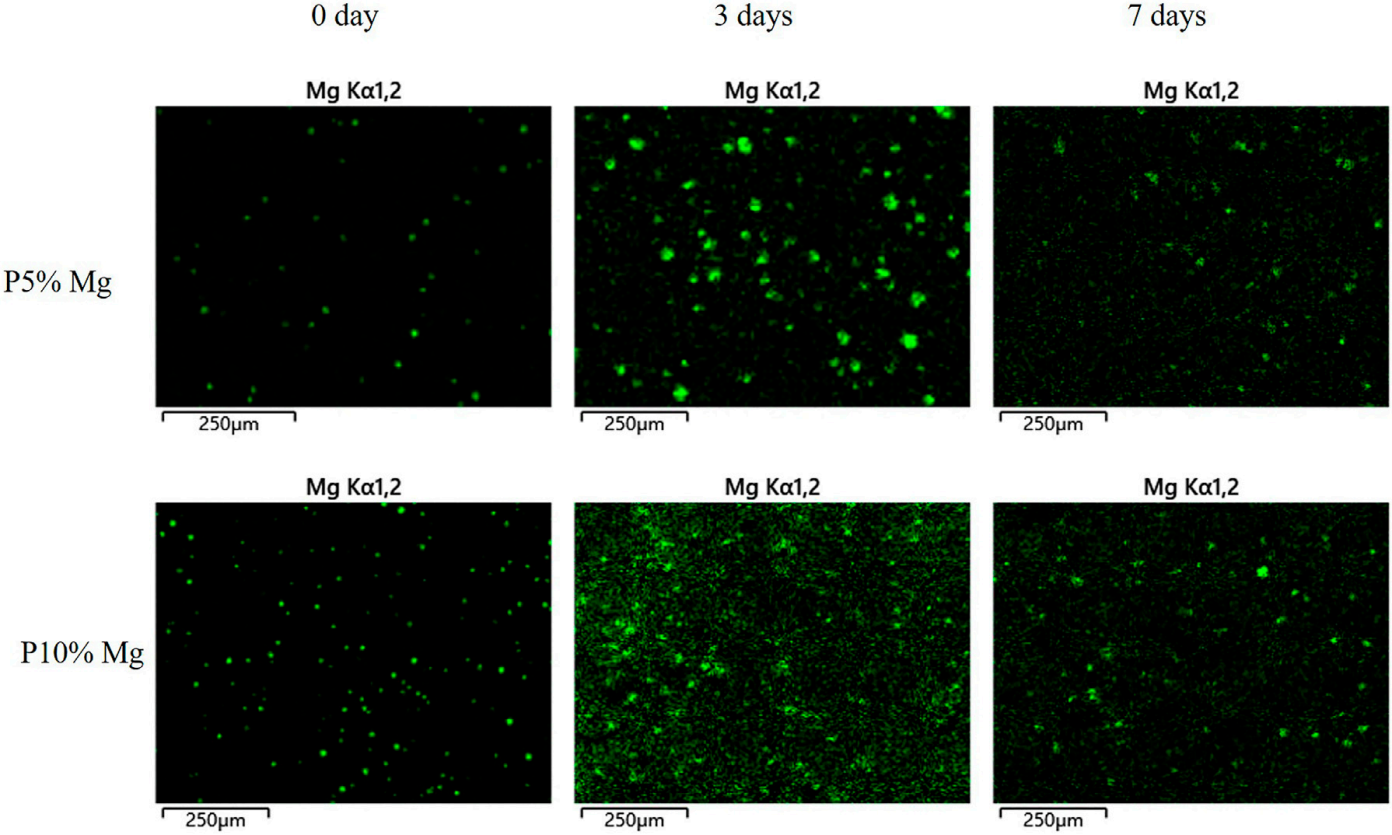
EDS-mapping analysis of the surfaces of P5% Mg and P10% Mg for 0, 3, and 7 days.

Distribution of magnesium particle in the composite with different culture time was determined using scanning electron microscope (SEM, Zeiss, Germany) coupled with energy dispersive spectrum (EDS) (Figure 2). The green fluorescence pots represented magnesium element. It indicated that magnesium element had been added to the composite material to distribute regularly on the surface at 0 day and continuously played important role over time. More magnesium element could be detected on the P10% Mg group. At the time point of 3 and 7 days, magnesium element could be observed which indicated that magnesium degraded gradually during the culture time. The results confirmed the conclusion of constant degradation of magnesium element.

### Immersion Test for pH Value Monitoring

**FIGURE 3:**
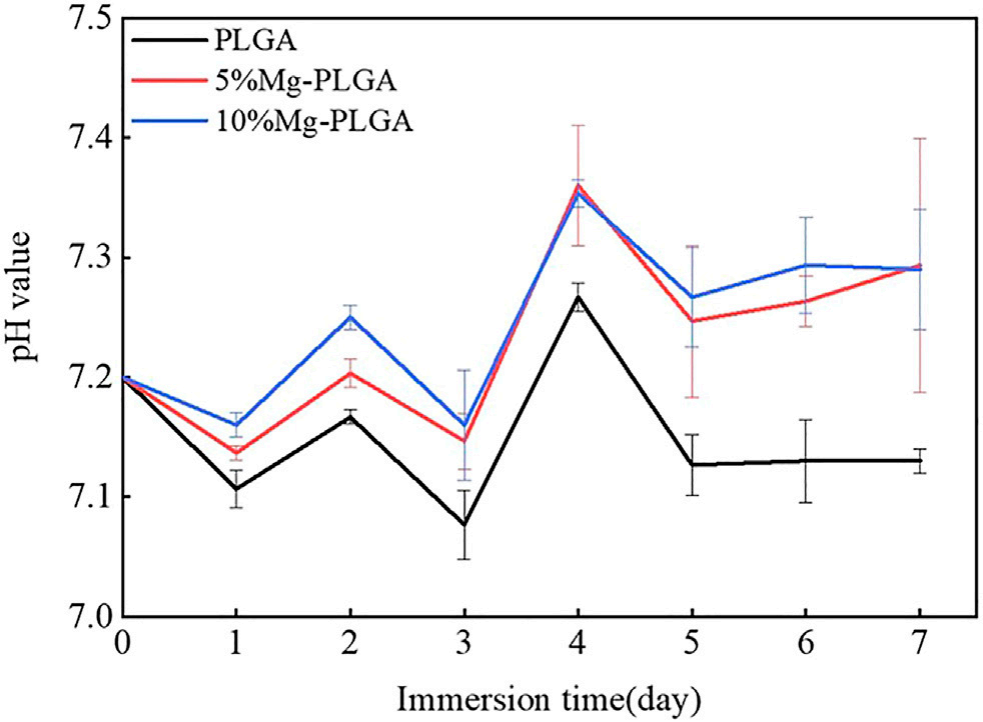
Results of pH value monitoring. The peak value of three groups appeared on the fourth day. From the fifth day on, the pH value of three groups leveled out gradually. With the increasing of the ratio of the magnesium, the pH value increased correspondingly between the P5% Mg group and P10% Mg group. The average value of the P10%Mg group was closer to the normal physiological value.

The pH value of the normal physiological environment was between 7.35 and 7.45. Figure 3 shows the results of the pH monitoring. The general trend from day 1 to day 7 was consistent among the three groups. For the PLGA group, the peak value, which was lower than the normal value, appeared on the fourth day, and the average value was lower than that of the P5% Mg and P10% Mg groups. With the increase in the ratio of Mg, the pH value increased correspondingly between the P5% Mg and P10% Mg groups. The average value of the P10% Mg group was closer to the normal physiological value. From the fifth day onwards, the pH values of the three groups leveled out gradually.

### *In Vitro* Experiments

#### Cell Cytotoxicity

**FIGURE 4:**
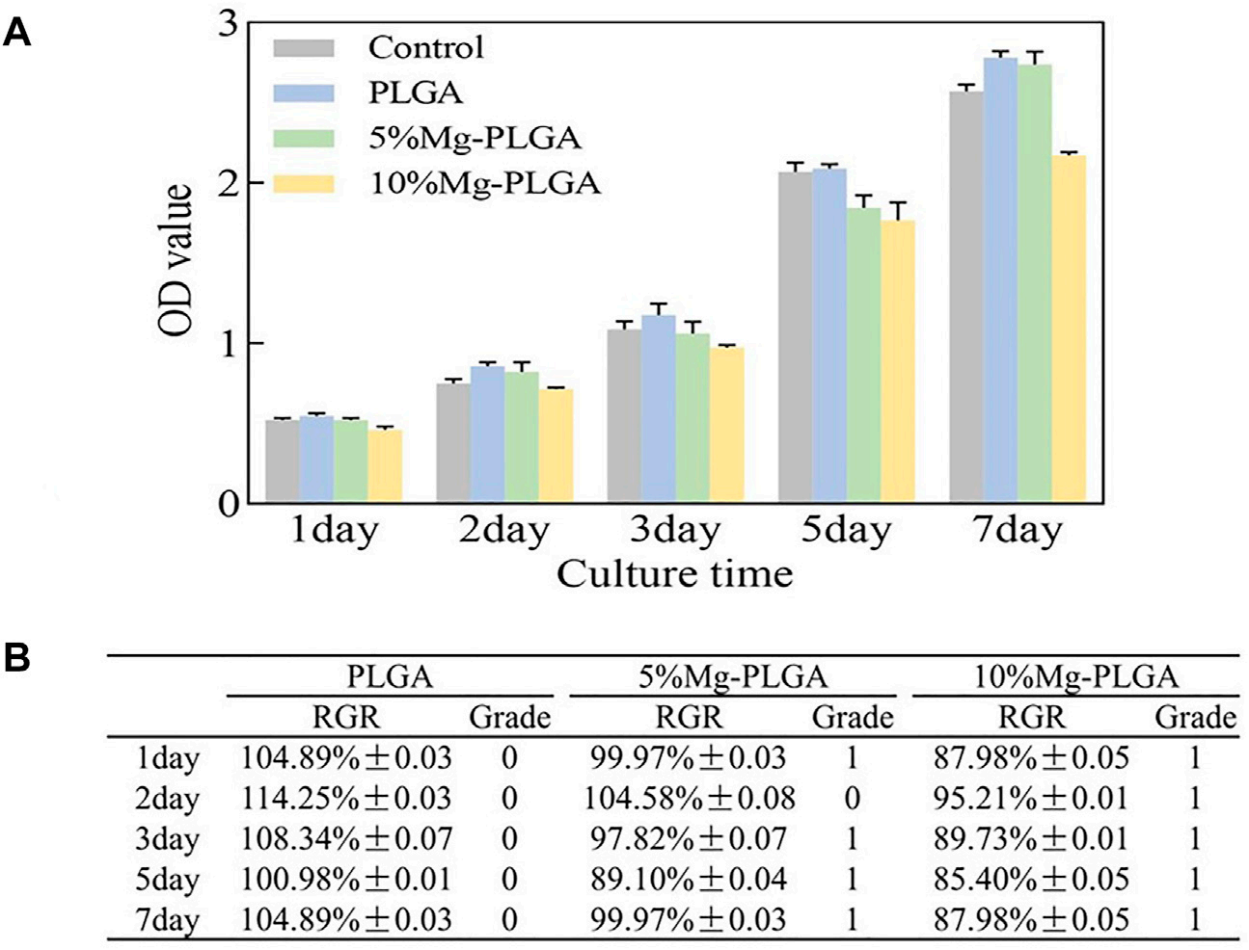
Results of cell proliferation and cell cytotoxicity. **(A)** The results of the proliferation of MC3T3-E1 cells cultured with samples. The OD value of all the groups gradually increased over time. There were no statistically significant for the differences between groups (*p* < 0.05). **(B)** The results of the cell toxicity grade (CTG). The CTG of the P group, P5% Mg group, and P10% Mg group showed 0, 0 or 1, and 1, respectively according to the standard United States Pharmacopeia. It indicated that all the samples showed no toxicity to cells.

Figure 4A shows the proliferation of MC3T3-E1 cells cultured with samples. The OD values of all groups gradually increased over time. No statistically significant differences were found between the groups (*p* < 0.05). Figure 4B shows the results for the CTG. For the P group, the average RGR value was greater than 100 from day 1 to day 7. For the P5% Mg and P10% Mg groups, the average RGR value was greater than 80 from day 1 to day 7, except 104.58% ± 0.08, which appeared on the second day in the P5% Mg group. The CTGs of the P, P5% Mg, and P10% Mg groups were 0, 0, 1, and 1, respectively. None of the samples showed toxicity to the cells.

#### Cell Apoptosis

**FIGURE 5:**
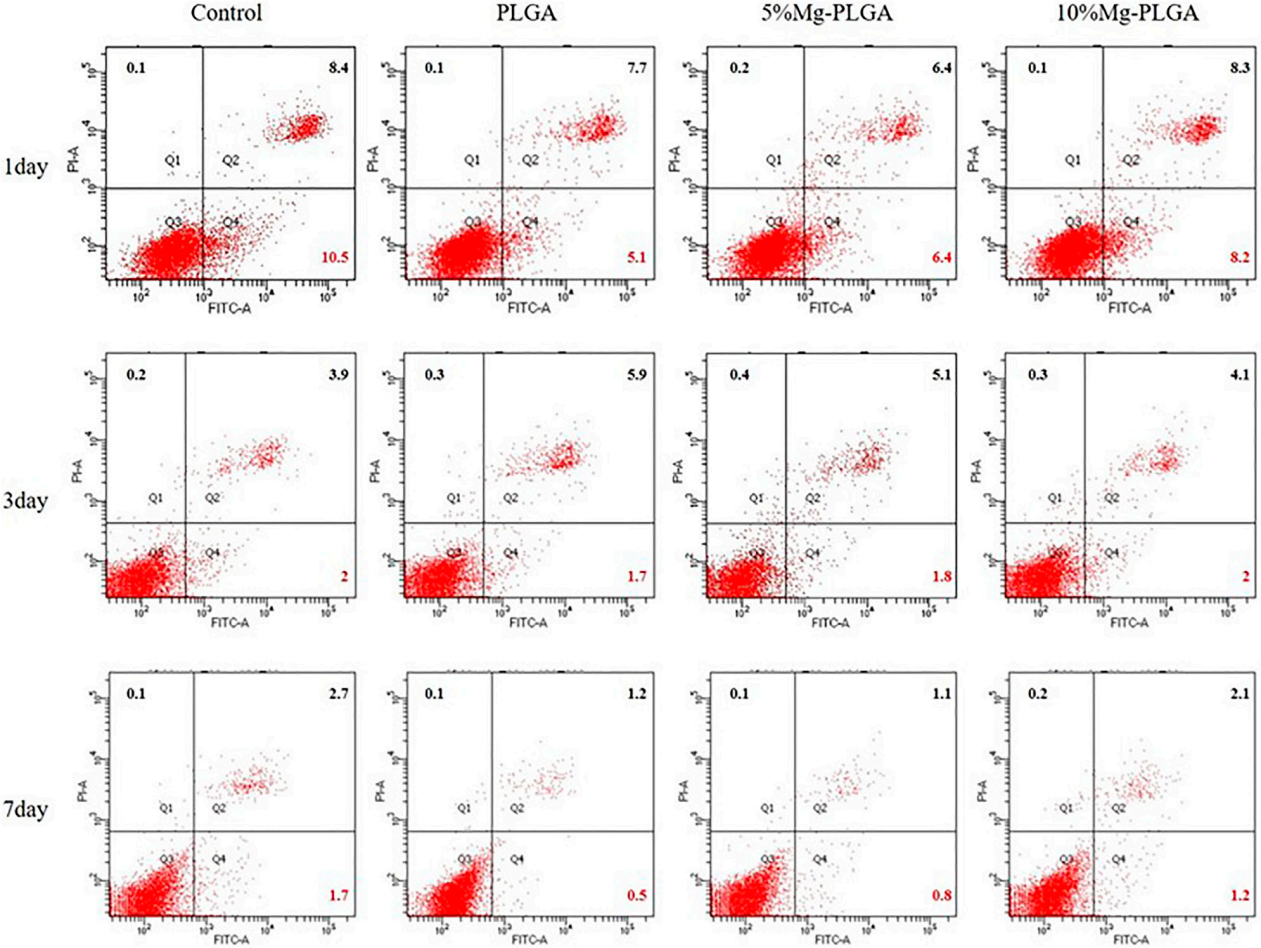
The apoptosis rates of MC3T3-E1 cells cocultured with the samples for 1, 3, and 7 days. Early apoptotic, dead and late apoptotic cells were localized in the lower right (Q4), upper left (Q1), and upper right (Q2) quadrant of a dot-plot graph respectively. It showed that with the increasing of the ratio of the magnesium, the early apoptosis rate of P5% Mg group and P10% Mg group increased correspondingly compared with P group at each time point, but lower than control group. The rates of the early apoptotic cells were basically proportional to the ratio of the magnesium.

The scatter plot of flow cytometry is shown in Figure 5. It shows the results of the apoptosis rates of MC3T3-E1 cells co-cultured with samples for 1, 3, and 7 days. Early apoptotic, dead, and late apoptotic cells were localized in the lower right, upper left, and upper right quadrants of a dot-plot graph, respectively. The early apoptosis rates of the negative, P, P5% Mg, and P10% Mg groups after 1 day in co-culture were 10.5, 5.1, 6.4, and 8.2%, respectively. After 3 days in co-culture, the early apoptosis rates of the control, P, P5% Mg, and P10% Mg groups were 2, 1.7, 1.8, and 2%, respectively. After 7 days in co-culture, the early apoptosis rates of the negative, P, P5% Mg, and P10% Mg groups were 1.7, 0.5, 0.8, and 1.2%, respectively. With the increase in the ratio of Mg, the early apoptosis rates of the P5% Mg and P10% Mg groups increased correspondingly compared with the P group, but lower than that of the control group. The rates of early apoptotic cells were proportional to the ratio of Mg.

#### Cell Adhesion

**FIGURE 6:**
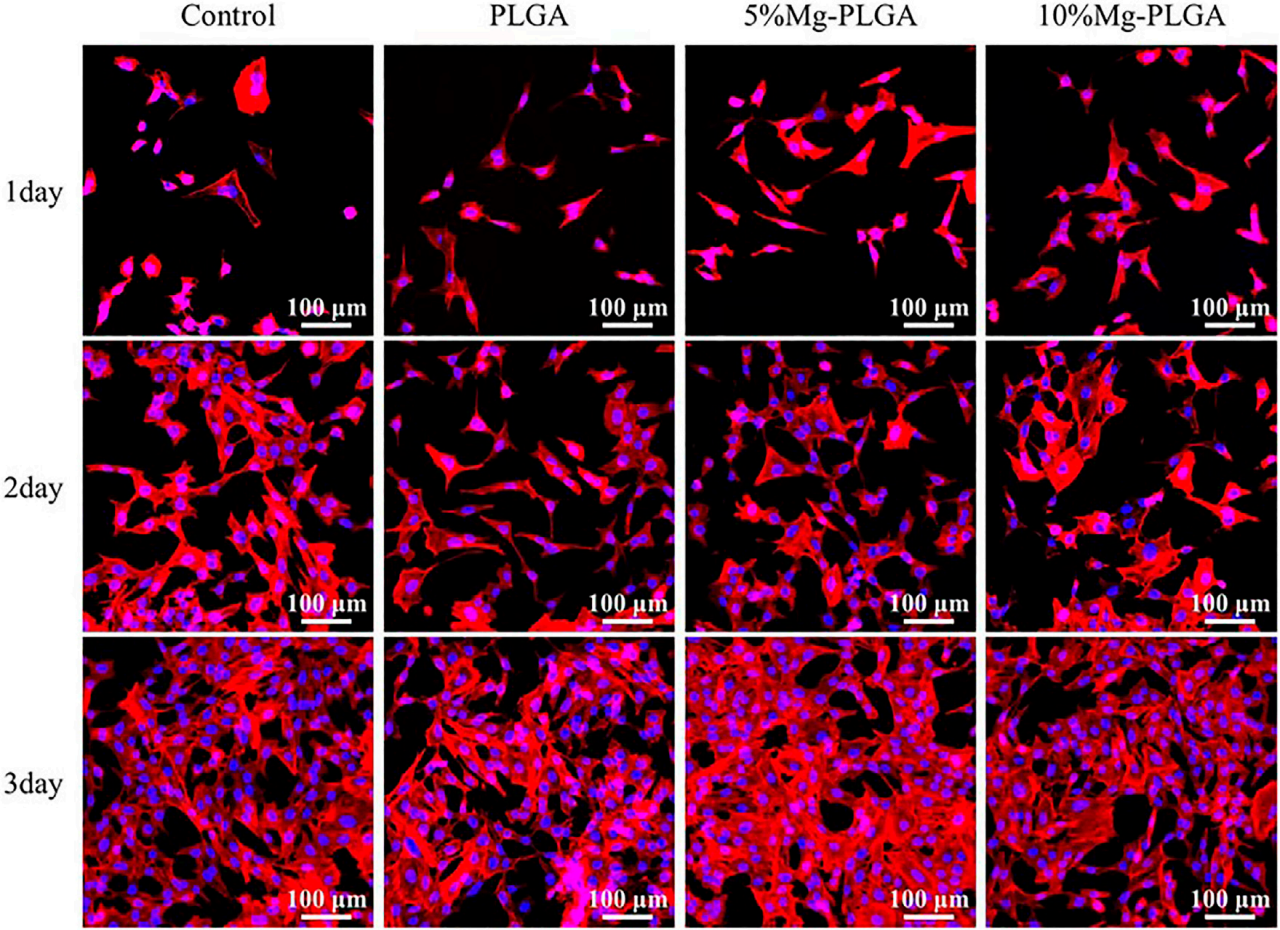
It showed the cytoskeletons and the nucleus of the MC3T3-E1 cells cultured on samples for 1, 2, and 3 days. It indicated that there was no significant difference for cell shape and number between groups. The samples in P group, P5% Mg group, and P10% Mg group were favorable to initial attachment and spreading of cells.

Figure 6 shows the cytoskeletons and nuclei of MC3T3-E1 cells cultured with samples for 1, 2, and 3 days. The cytoskeletal fluorescence was stained with phalloidin, and the nucleus was stained with DAPI. No significant difference was found for cell shape and number between groups, which was the same as the results of cell proliferation mentioned above. Therefore, it can be concluded that the samples in the P, P5% Mg, and P10% Mg groups were favorable for the initial attachment and spreading of cells. Finally, the results indicated that adding Mg at a ratio of less than 10% would not inhibit cell adhesion compared with the control P group.

#### ALP Activity Staining

**FIGURE 7:**
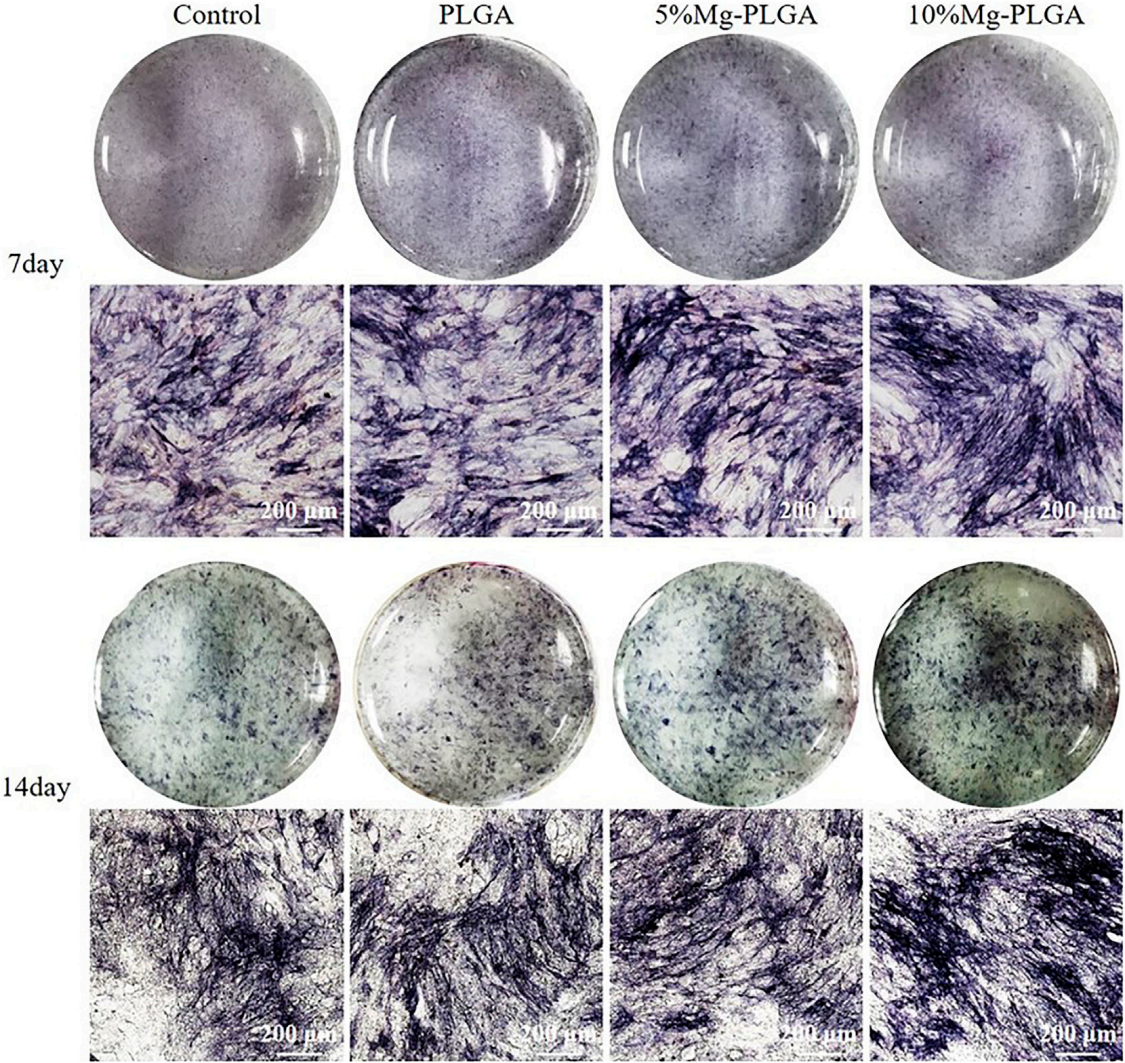
It showed the ALP staining of MC3T3 cells cultured with PLGA, PLGA with 5% magnesium, and PLGA with 10% magnesium for 7 and 14 days, respectively. With the increasing of the ratio of magnesium, ALP activity increased correspondingly.

Figure 7 shows the ALP staining of MC3T3-E1 cells cultured with PLGA, PLGA with 5% Mg, and PLGA with 10% Mg for 7 and 14 days. At 7 days after osteogenic induction, with the increase in the ratio of Mg, ALP activity increased gradually with increasing dark staining in the figure. In addition, no significant difference was found between the control and PLGA groups. The same trend was observed at 14 days after osteogenic induction.

## Discussion

Bone defects resulting from trauma, inflammation, and cancer are common therapeutic problems in the field of oral and maxillofacial surgery (Mardas et al., 2014). Autologous/allogenic grafting procedures are usually performed as an effective method to complete bone repair in clinical practice (Wei et al., 2020). However, the most common disadvantage after the procedure is injury to the donor site. In recent years, biomaterials have been accepted as a potential alternative to standard autologous/allogenic grafting procedures to achieve clinically successful bone regeneration (Thrivikraman et al., 2017). Among the biomaterials, PLGA as a biodegradable polyester has received attention because of its excellent biocompatibility and tunable physicochemical properties. It has been used to produce biodegradable sutures and orthopedic fixation devices for surgical procedures (Middleton and Tipton, 2000). However, the acidic products produced during the degradation of PLGA limit its widespread use as a bone repair material. Mg, as a metallic biomaterial, possesses numerous advantages, such as biodegradability, biocompatibility, and mechanical properties, similar to those of bone. However, its disadvantages include low corrosion resistance against living body conditions, rapid loss of mechanical integrity, hydrogen evolution, and alkalization during degradation (Ding, 2016). Therefore, the current study aimed to combine the physicochemical properties of PLGA and Mg to develop a composite biodegradable material. Some researchers have reported the application of PLGA combined with Mg by coating PLGA on the surface of Mg or 3D printing synthetic material (Brown et al., 2015; Liu et al., 2015). In contrast to the above reports, we improved the forming method of materials and examined cell cytotoxicity, cell adhesion, and osteogenic properties.

### pH Value Monitoring

The degradation products of pure Mg included H_2_ gases and released ions (Mg^2+^, OH^−^) obeying the following formula (Amukarimi and Mozafari, 2021; Jana et al., 2021):

$$\text{Mg}\to {\text{Mg}}^{2}+2\text{e}$$

$${2\text{H}}_{2}\text{O}+2\text{e}\to {2\text{OH}}^{-}+\text{H}2$$

$${\text{Mg}}^{2}{+2\text{OH}}^{-}\to \text{Mg}{\left(\text{OH}\right)}_{2}$$

The major degradants of PLGA were oligomers, such as LA and GA, at the initial stage, while displaying an initial increase in pH. Li et al. reported that the degradable oligomers for PLGA 50 were L_4_G_2_ oligomers and for PLGA 80 were G_10_ and L_7_at the initial stage (Li et al., 2018). As degradation proceeded, we proposed that the formation of basic Mg(OH)_2_ through Mg degradation would react with the acidic byproducts of PLGA to complete pH neutralization and promote osteogenic osteogenesis. The results of our study confirmed this hypothesis. The increase in pH can be attributed to the degradation of exposed Mg particles because these particles spontaneously react with water to produce Mg hydroxide (Pogorielov et al., 2017). We then observed subsequent neutralization resulting from acidic degradation of PLGA. In the current study, the 10 wt% Mg group maintained a pH value closer to the normal value. Therefore, with the increase in the ratio of Mg in the composite material, the reaction time of the Mg and acidic byproducts of PLGA would be longer. The microenvironment of the composite biomaterials with 10 wt% Mg maintained a longer neutralized status and possessed better osteogenesis than 5 wt% Mg.

### *In Vitro* Experiment

In the current study, the cytocompatibility of the PLGA/Mg composite biodegradable material was investigated using cell proliferation and cytotoxicity tests. The results of the cell proliferation assay showed no significant difference between the groups, and the composite biodegradable material would not be deleterious to cell viability. The same trend was observed in the cell adhesion assay. These results are in accordance with Zhao et al.’s conclusions. Zhao et al. reported PLA/Mg composites for orthopedic implants and evaluated the influence of Mg on the degradation and biocompatibility of PLA. The cytotoxicity results showed that the PLA/Mg composites exhibit no toxicity in osteoblastic cell culture (Zhao et al., 2017). However, by summarizing the data of the CCK-8 test, the average OD values of the composite biodegradable material groups were lower than those of the PLGA and control groups and higher than those of the PLGA with 5 wt% Mg group and PLGA with 10 wt% Mg group. These findings could be due to the formation of hydrogen gas pockets within the composite materials during the degradation of Mg, which inhibited the proliferation of cells, and the amount of gas pockets increased with the increase in the ratio of Mg. Therefore, the ratio of Mg was controlled to less than 10% in the current study. Sayuri et al. reported that the cells proliferate the fastest *in vitro* after the addition of 10 mM Mg^2+^, but cell proliferation is inhibited at higher concentrations (Mg^2+^ > 20 mM). This cytotoxicity has also been reported for other metal ions (Na, Cr, Mo, Al, Ta, Co, Ni, Fe, Cu, Mn, and V) in osteoblasts (Hallab et al., 2002; Yoshizawa et al., 2014).

Programmed cell death is called apoptosis, which is a normal physiological process of cell death. Apoptosis process is controlled by genes, which are important for maintaining the stability of the intracellular environment (Chen et al., 2014; Shih et al., 2015). Researchers usually use the apoptosis rate to reflect the ability of cells to adapt to different surfaces. The apoptosis rate reflects the state of cells on the material surface. Flow cytometry is now a widely used method for analyzing the expression of cell surface and intracellular molecules (Bolton and Roederer, 2009; Maecker, 2009; Zaritskaya et al., 2010). Our current data showed that the early apoptosis rate in the lower right quadrants in the three groups was lower than that in the control group, but the rate increased significantly among the experimental groups with the increase in Mg concentration at different time points. The possible reasons include the existence of mental Mg particles that induce cell apoptosis. The other reason could be attributed to oxidative stress response during Mg hydroxide formation on the material surface, promoting osteoblast apoptosis.

In the current study, the data of ALP activity staining and q-PCR supported the conclusion that higher Mg concentrations promote osteogenic regeneration. However, with the increase in the concentration of Mg, cell proliferation is inhibited. Hence, Mg ion concentration should be controlled within a certain range. This was the reason why the quality ratio of Mg in the composite material did not exceed 10 wt%. Our results are consistent with those reported by other researchers. Xu (Xu et al., 2018) added Mg metal and Mg alloy to PLGA samples to develop scaffold materials. The effects of material osteogenesis were evaluated using ALP expression and cellular mineralization, and the Mg and Mg alloy scaffolds were concluded to outperform the control PLGA film.

These two factors could contribute to osteogenesis. On one hand, self-neutralizing ability provided a favorable environment for the osteogenic regeneration of cells. Acidic decomposition products of PLGA are known to induce inflammatory reactions (Ji et al., 2012; Washington et al., 2018). The products of Mg degradation react with the acidic byproducts of PLGA, lactic acid, and glycolic acid, leading to pH neutralization. On the other hand, Mg^2+^ ions stimulate intracellular signaling pathways that may enhance mineralization and bone regeneration. Ca^+2^ ions contribute to osteogenic differentiation (Dvorak et al., 2004; Dvorak and Riccardi, 2004; Nakamura et al., 2010). We speculated that Mg^2+^may act through a similar activation cascade to induce osteogenic regeneration *via* the activation of a specific transcription factor. Insulin-like growth factor 2 plays an important role in long bone growth, and its upregulation by Mg ions is indicative of the effect of Mg on bone growth (Fisher et al., 2005). Other researchers have reported that Mg^2+^ ions can upregulate Akt phosphorylation to promote ALP activity and enhance the expression of osteogenesis-related genes (Wang et al., 2017). These conclusions confirm our results.

In conclusion, PLGA with an Mg composite biodegradable material demonstrated favorable cytocompatibility and osteogenesis in MC3T3-E1 cells. Compared with PLGA with 5 wt% Mg and PLGA groups, PLGA with 10 wt% Mg possessed effective osteogenic properties and showed no toxicity to cells. Therefore, the novel composite material could provide a wide range of applications in bone defect repair and scaffold-based tissue engineering in clinical practice.

## Data Availability

The raw data supporting the conclusion of this article will be made available by the authors, without undue reservation.
